# Exploring mindfulness interventions across cultures: a comparative meta-analysis of mindfulness interventions for athletes in Western and Eastern contexts

**DOI:** 10.3389/fpsyg.2024.1449886

**Published:** 2024-10-31

**Authors:** Senyao Du, Ziheng Ning

**Affiliations:** Faculty of Health Sciences and Sports, Macao Polytechnic University, Macau, Macao SAR, China

**Keywords:** meta-analysis, mindfulness, acculturation, cultural adaptation, sports performance, psychological flexibility

## Abstract

**Objective:**

Applying mindfulness training across cultures can be challenging because its internal motivation depends on values, but there are differences between Western and Eastern contexts. This meta-analysis examines the comparative impacts of the Western Mindfulness-Acceptance-Commitment (MAC) approach and the culturally-adapted Chinese Mindfulness-Acceptance-Insight-Commitment (MAIC) mindfulness training for athletes, to explore the importance of cultural adaptation in cross-cultural psychology to psychological skills training.

**Methods:**

This meta-analysis was registered with Prospero (ID: CRD42023474474) and adhered to the PRISMA principles. The PICO search methodology was employed to identify pertinent Chinese and English literature across six databases (Web of Science, PubMed, Scopus, CNKI, Wanfang, and VIP) from 2003 to 2023. The evidence originated from randomized controlled trials. Effect sizes were evaluated across three primary domains: athletic performance, mindfulness, and psychological flexibility, and heterogeneity and publication bias.

**Results:**

Both MAC and MAIC demonstrated positive effects across all outcome categories in 18 studies. MAIC exhibited substantially larger effect sizes compared to MAC. In mindfulness (MAIC: *g* = 1.273, *p* < 0.001; MAC: *g* = 0.521, *p* = 0.001), sports performance (MAIC: *g* = 0.945, *p* = 0.015; MAC: *g* = 0.550, *p* < 0.001), MAC did not provide evidence of improved psychological flexibility (*g* = 0.076, *p* = 0.747), while MAIC demonstrated significant benefits (*g* = 0.964, *p* < 0.001).

**Discussion:**

While MAIC is based on MAC, it yields more significant outcomes. This may be the “Insight” component introduced by MAIC, which skillfully integrates Eastern context. The MAIC strategy not only becomes more appropriate for local athletes but also indirectly enhances their mindfulness, athletic performance, and psychological flexibility, resulting from cultural adaptation. The new idea not only solves the culture shock caused by the direct application of Western MAC approach on Chinese athletes, but also provides a new solution to the problem that there is insufficient evidence to support the effect of the MAC approach on “psychological flexibility.” Investigating differential effects between MAC and MAIC contributes to understanding the significance of culturally adapting mindfulness training and transnational movement of practices. The findings will assist practitioners in selecting evidence-based protocols tailored to athlete needs and cultural contexts.

**Systematic review registration:**

Prospero (ID: CRD42023474474).

## 1 Introduction

Psychological skills training (PST) techniques, such as imagery and self-talk, have been widely recognized for their potential to enhance athletic performance by stabilizing methods and optimizing competitive mentality (Siekańska et al., 2021). In recent years, mindfulness-based training has emerged as a promising approach for athletes. Rooted in Eastern meditative traditions, mindfulness interventions were introduced to Western clinical contexts by Kabat-Zinn in 1979 and later adapted specifically for athletes to develop a series of mindfulness training specifically for athletes (Kabat-Zinn, 2003), such as: Mindfulness-Acceptance-Commitment approach (MAC, Gardner and Moore, 2004; Moore, 2009); Mindful-Sport-Performance-Enhancement (MSPE, Kaufman et al., 2009); Mindfulness-Meditation-Training for Sport (MMTS, Baltzell and Summers, 2018).

Recognizing the limitations of traditional PST, Gardner and Moore (2004) developed the Mindfulness-Acceptance-Commitment (MAC) approach, which integrates acceptance commitment therapy (ACT, Hayes and Pierson, 2005) and mindfulness-based cognitive therapy (MBCT, Segal et al., 2002). PST emphasizes improving internal states and will may inadvertently increase unwanted internal states. This is called Ironic effects occurring after thought suppression, also known as rebound effects (Wang et al., 2021). Unlike that, MAC emphasizes acceptance and “Wu-wei (inaction),” allowing psychological experiences to arise and pass naturally (Kee et al., 2021).

Over the past two decades, Gardner and Moore have continually refined and evaluated MAC (Gardner and Moore, 2004, 2012; Moore, 2009). A recent meta-analysis revealed that MAC enhanced athletes’ mindfulness (*g* = 0.64, 95% CI [0.00, 0.91]) and potentially sports performance (*g* = 0.37, 95% CI [0.00, 0.79]), but its effects on well-being and psychological flexibility remain inconclusive (Ptáček et al., 2023). Multiple studies demonstrate MAC’s efficacy in improving mental health outcomes, including anxiety, depression, and self-compassion (Gross et al., 2018; Oguntuase and Sun, 2022; Sabzevari et al., 2023). Moreover, athletes who engage in mindfulness can improve their focus on themselves, perhaps reducing accidents and promoting physical health (Naderi et al., 2020; Zadeh et al., 2019).

However, problems arise when using MAC effectively in different socio-cultural contexts (such as “National System” for China’s competitive sports). MAC aims to develop mindful present-moment acceptance of internal experiences, clarification of valued goals, and response to external cues, Reactions and attention to emergencies are used to improve sports performance. Internal motivation mostly derives from individual values (Gardner and Moore, 2012), while socially oriented values are the more dominant way of thinking in Chinese society, and research shows that when MAC is applied to Chinese Sanda and Wushu athletes, it is difficult for them to recognize or clarify their own values from socially oriented values (Bu and Si, 2014). Furthermore, even when athletes recognize their individual values, they have difficulty realizing those values. It is believed that these difficulties are related to Chinese culture and its unique sports system (Si et al., 2014). They may conflate social values with individual values, thereby disregarding their true desires, complicating their understanding of their internal state and exterior behavior, which undermines the efficacy of mindfulness approach. In order to better utilize the training methods born in different cultural backgrounds, it is necessary to solve the impact of this different culture (Roychowdhury et al., 2021; Su et al., 2019).

Research on transnational migration has flourished in recent years, but transnational movement of ideas and practices has received little attention (Roychowdhury et al., 2021). Acculturation plays a pivotal role in the field of cross-cultural psychology, referring to the phenomenon wherein two distinct cultural groups engage in direct and continuous interaction, leading to alterations in the original cultural patterns of one or both parties (Berry, 2003; Yu and Zheng, 2005). MAC, originating from Eastern Buddhism, yielded positive outcomes when developed and implemented by Western researchers, but faced difficulties when directly applied to Eastern athletes (Bu and Si, 2014). This practice has undergone subtle changes due to cultural adaptation during the ongoing exchange between Eastern and Western cultural contexts. It is crucial to acknowledge that adopting intervention methods from diverse cultural backgrounds without incorporating necessary cultural adaptations may introduce bias into the effectiveness of such interventions. In response to this concern, Chinese scholars have developed an adapted version of the Western-derived MAC approach known as Mindfulness-Acceptance-Insight-Commitment (MAIC), It was developed on the basis of MAC and combined with Chinese social and cultural factors (i.e., acceptance-based coping with adversity, socially oriented values and insights). Which effectively integrates Eastern values (Su et al., 2019).

Given mindfulness’s Eastern origins, aligning training with Eastern mental frameworks is expected to enhance its effectiveness for Chinese athletes. Built upon MAC, MAIC integrates “Insight” cultivation to promote dialectical thinking, aligning with China’s collectivist context. As athletes navigate personal growth within social duties (Si and Jiang, 2011). Insight enables flexibility in perception (Öllinger and Knoblich, 2009), the addition of “Insight” may support self-liberation and self-sublimation in the face of adversity (Si et al., 2014). Ultimately, localized approaches like MAIC may better resonate with Chinese athletes. Although MAIC has only been implemented for less than 10 years, studies evaluating MAIC training for Chinese athletes provide initial evidence of positive effects on mindfulness, sports performance, health, and related psychological components (Feng and Si, 2015; Bu, 2015; Bu and Si, 2014).

Notably, while MAC fails to present compelling evidence for enhancing psychological flexibility, emerging findings suggest that MAIC enhances this critical capacity for managing adversity (Zhang et al., 2016; Bu et al., 2020). However, considerable debate surrounds the effects of mindfulness on positive emotions, well-being, and psychological flexibility (Liu et al., 2013; Xu et al., 2015). A prior meta-analysis examined the effects of MAC (Ptáček et al., 2023), nonetheless, potential variations in the MAC approach following its revision and adaptation to Chinese culture were not taken into account (Zhang et al., 2016).

While sharing a common foundation, differences between the Western-derived Mindfulness-Acceptance-Commitment (MAC) and the culturally-adapted Mindfulness-Acceptance-Insight-Commitment (MAIC) approaches may exert influence on outcomes. As globalization progresses, psychological skills training is evolving via the integration of the cultural of Eastern and Western contexts. It is essential to consider the disparities that may arise from varying cultural backgrounds during borrowing and transferring, it becomes imperative to systematically evaluate various approaches, taking into account cultural nuances. And the recently developed MAIC approach tailored for Chinese athletes necessitates further validation (Zhang et al., 2016; Bu, 2015). Rigorous evaluation is indispensable in substantiating preliminary indications of localization benefits.

To verify the significance of cultural adaptation in practices, this study explored cross-cultural mindfulness interventions through a meta-analysis approach, aims to: (1) inductively synthesize available data from the past two decades, (2) quantitatively compare outcomes associated with the Western-derived MAC and the culturally-adapted MAIC approach, and (3) offer insights for future culturally-adapted applications. This meta-analysis did not consider the inclusion of other mindfulness training programs (such as MSPE and MMTS). The main reason is that the difference between MAC and MAIC is only the improvement of local cultural adaptation. There is still a big difference in the actual operation of the others mindfulness trainings. Ensure the quality of meta-analysis, reduce bias to a certain extent, and more directly explain the impact of cultural adaptation. We hypothesize that the culturally adapted MAIC approach will more effectively improve psychological flexibility, mindfulness levels, and sports performance outcomes among Chinese athletes compared to the standard MAC approach. Investigating differential effects between MAC and MAIC contributes to understanding the significance of culturally adapting mindfulness training and transnational movement of practices. The findings will assist practitioners in selecting evidence-based protocols tailored to athlete needs and cultural contexts.

## 2 Methods

### 2.1 Search strategy and study selection

This meta-analysis research has been registered on Prospero (ID: CRD42023474474) and follows the latest version of the Preferred Reporting Items for Systematic Reviews and Meta-Analyses (PRISMA) guidelines (Page et al., 2021). To ensure the inclusion of high-quality and sufficient empirical studies, multiple searches and inspections were conducted. The PICO search strategy was employed to determine the appropriate search terms, considering the population (athletes), intervention (MAC or MAIC), comparison (control group or pre-post intervention), and outcomes (all outcomes that may be influenced by MAC and MAIC, comprises four dimensions: mindfulness, sports performance, health, and mindfulness-related psychological components).

The first author and a researcher independently searched for relevant literature published in Chinese and English from 2003 to 2023 in six databases: Web of Science, PubMed, Scopus, CNKI, Wanfang, and VIP. These databases were selected due to their comprehensive coverage of peer-reviewed journals in the fields of psychology, sports science, and complementary and alternative medicine.

For Chinese literature, “advanced search” and “specific queries” were utilized in CNKI, Wanfang, and VIP databases. The search terms included “mindfulness training” OR “Mindfulness-Acceptance-Insight-Commitment” OR “MAIC” OR “Mindfulness-Acceptance-Commitment” OR “MAC” AND “athlete” (in Chinese). The “article abstract” function was used in CNKI, while the “title and keywords” feature was employed in Wanfang and VIP. Due to VIP database limitations, only a maximum of 5 advanced filtering terms could be entered; consequently, “MAC” and “MAIC” were removed from the search in this database.

For English literature, the search formula “(((mindfulness) OR (mindfulness-acceptance-insight-commitment) OR (mindfulness-acceptance-commitment)) AND ((athlete*) OR (player*)))” was used in Web of Science, PubMed, and Scopus. This comprehensive search strategy aimed to capture all relevant studies investigating the effects of MAC or MAIC on athletes’ outcomes.

In addition to the database searches, the reference lists of included articles and relevant systematic reviews and meta-analyses were manually searched to identify any additional eligible studies. Furthermore, the authors conducted a gray literature search, including dissertations and conference proceedings, to minimize the risk of publication bias.

The study selection process is presented in a PRISMA flow diagram, depicting the number of studies identified, screened, and included in the meta-analysis. This transparent reporting of the search and selection process enhances the reproducibility and reliability of the findings.

### 2.2 Inclusion and exclusion criteria

The two authors collaboratively established the inclusion and screening criteria, which were subsequently used by the first author to independently assess and eliminate entries that did not meet these criteria. The inclusion criteria were as follows:

1. Study design: Only randomized controlled trials (RCTs) were included to ensure the highest level of evidence and minimize the risk of bias. Specifically, setting up a control group (control), randomization of research subjects (Randomization) and blind trial (blind) are necessary.
2. Intervention: The study must have investigated the effects of either the Mindfulness-Acceptance-Commitment (MAC) approach or the Mindfulness-Acceptance-Insight-Commitment (MAIC) approach. The intervention had to follow the MAC protocol as described by Gardner and Moore (2004) or the MAIC “Mindfulness Training Manual for Chinese Athletes” developed by Si et al. (2014).
3. Participants: Studies involving athletes of any age, gender, skill level, and sport type were included. Both individual and team sports were considered.
4. Outcomes: The study had to report at least one of the following outcomes: mindfulness, sports performance, health (including physical and mental health measures), or mindfulness-related psychological components (such as psychological flexibility, flow, or attention).
5. Language: Studies published in either English or Chinese were included to ensure a comprehensive review of the available literature.
6. Publication status: Both published articles in peer-reviewed journals and unpublished dissertations were considered to minimize the risk of publication bias.

Studies were excluded if they met any of the following criteria:

1. Non-compliance with the inclusion criteria, such as non-RCT study designs, interventions not following the MAC or MAIC protocol, or studies not reporting relevant outcomes.
2. Animal studies were excluded as the focus of this meta-analysis was on human participants.
3. Conference abstracts, book chapters, and literature reviews were excluded due to the lack of detailed information required for meta-analysis.
4. Studies with missing or duplicated data were excluded to ensure the accuracy and reliability of the analysis.
5. Pre-experiments, pilot studies, and quasi-experimental research were not included to maintain the focus on high-quality RCTs.

The strict adherence to these inclusion and exclusion criteria ensured that only relevant, high-quality studies were included in the meta-analysis, minimizing the risk of bias and enhancing the validity of the findings. Any discrepancies in the application of the criteria were resolved through discussion and consensus among the authors.

### 2.3 Study quality assessment

To ensure the rigor of this meta-analysis, the first author and the researcher independently assessed the quality of the selected RCT studies using the PEDro scale (Sherrington et al., 2000). The PEDro scale is a widely accepted tool for evaluating the methodological quality of RCTs, It possesses moderate to high reliability in assessing RCTs (Maher et al., 2003). It is consisting of 11 items that assess various aspects of study design, implementation, and reporting. Each item is scored as either present (1 point) or absent (0 points), with a total score ranging from 0 to 10 (item 1 is not included in the total score).

Studies were classified as high quality (8–10 points), moderate quality (4–7 points), or low quality (0–3 points) based on their PEDro scores. The two researchers conducted the quality assessment independently and without communication to minimize bias. Disagreements were resolved through discussion and consensus, with the involvement of a third researcher when necessary.

After the quality assessment, a Kappa consistency test (Cohen’s Kappa) was performed to evaluate the inter-rater agreement between the researchers. The Kappa coefficient ranges from −1 to 1, with values above 0.6 indicating substantial agreement. This test helps to ensure the reliability of the quality assessment process and the overall robustness of the meta-analysis.

Studies of low quality were excluded from the meta-analysis to maintain the integrity of the findings, while studies of moderate and high quality were included. This approach ensures that the conclusions drawn from the meta-analysis are based on the best available evidence from well-designed and well-conducted RCTs.

### 2.4 Data extraction

Following the screening and exclusion process, the selected literature was meticulously examined, and pertinent data were extracted using a standardized data extraction form developed by the authors. The extracted data included:

1. Study characteristics: title, publication year, first author, country, and funding source.
2. Participant characteristics: sample size, age, gender, sport type, skill level, and years of experience.
3. Intervention details: type of mindfulness training (MAC or MAIC), intervention duration, frequency, session length, and delivery format (e.g., group or individual sessions).
4. Control group details: type of control (e.g., waitlist, active control), and any interventions received.
5. Outcome measures: mindfulness, sports performance, health (physical and mental), and mindfulness-related psychological components (e.g., psychological flexibility, flow, attention). The specific measurement tools used for each outcome were also recorded.
6. Results: means, standard deviations, and sample sizes for each outcome measure at baseline and post-intervention for both the experimental and control groups. If available, follow-up data were also extracted.

The data extraction process was conducted independently by the first author and the researcher to ensure accuracy and completeness. Any discrepancies were resolved through discussion and consensus, with the second author. If the required data were not reported in the published article, the corresponding author was contacted via email to request the missing information. If the data remained unavailable after two attempts to contact the author, the study was excluded from the meta-analysis.

### 2.5 Data analysis

Data for each study, including sample sizes, means, standard deviations, *t*-values, and *p*-values, were input into Excel spreadsheets and subsequently entered into Comprehensive Meta-Analysis Version 3.0 (CMA) software for analysis. The effect size, represented by the standardized mean difference (SMD) and corrected using Hedges’ *g*, was utilized to examine the influence of mindfulness training on mindfulness, sports performance, health, and mindfulness-related psychological components. Hedges’ *g* provides a more accurate estimate of the effect size, especially for small sample sizes, by applying a correction factor to Cohen’s *d*. Effect sizes were interpreted as small (0.2), medium (0.5), and large (0.8) (Andrade, 2020).

The outcome variables were divided into four dimensions for integrated analysis: (1) mindfulness, (2) sports performance, (3) health, and (4) mindfulness-related psychological components. A random-effects model was employed, considering the potential variations in mindfulness training effects across individuals and sports. This model takes into account both within-study and between-study variability, providing a more conservative estimate of the overall effect size.

Heterogeneity among the included studies was assessed using the *I^2^* statistic, *Q* statistic and forest plots. Publication bias was evaluated using funnel plots and Egger’s linear regression test. Subgroup analyses were conducted to explore potential moderators of the effect sizes, such as participant characteristics, intervention characteristics, and type of control group. Sensitivity analyses were performed to assess the robustness of the findings by excluding studies with extreme effect sizes or high risk of bias, and by using alternative statistical models.

All statistical analyses were conducted using a significance level of 0.05, and 95% confidence intervals were reported for the effect sizes. The results of the meta-analysis were summarized using tables and figures, and interpreted in the context of the existing literature and the specific research questions addressed by this study.

## 3 Results

### 3.1 Search results and overview

A comprehensive search was conducted to identify relevant studies investigating the effects of mindfulness interventions on athletes. The initial search yielded a total of 1,859 potentially relevant documents, including 1,847 from electronic databases (Web of Science, PubMed, Scopus, CNKI, Wanfang, and VIP), nine from citation searches, and three from dissertations. After removing duplicates, 1,055 documents were screened for eligibility based on the predefined inclusion and exclusion criteria. The first author and the researcher independently assessed the titles, abstracts, and full texts of the identified articles, while the second author validated the selection process. Disagreements were resolved through discussion and consultation within another invited researcher when necessary.

Following the initial screening, 24 documents met the inclusion criteria and were selected for quality assessment. The quality of the selected articles was independently evaluated by two authors using the PEDro scale, the final average standard deviation of the PEDro ratings for the 24 articles was 7.42 ± 2.12, indicating a moderate to high level of overall quality. The Kappa consistency test was performed to assess the inter-rater agreement between the two authors, resulting in a coefficient of 0.719 (*p* < 0.001), suggesting a substantial level of agreement. Three low-quality documents were excluded based on the quality assessment (only three points), resulting in 21 documents eligible for data extraction. And the average standard deviation of the PEDro ratings for the 21 articles was 8.05 ± 1.38, indicating a high level of overall quality, quality has improved significantly. Of all the papers reviewed, only one article received a rating of 5.5 ± 0.5 from the first author and the researcher, while the remaining publications were graded between 6 and 11, all included publications satisfied the fundamental criteria for RCTs. Despite the potential bias introduced by medium-quality articles, the stringent inclusion and exclusion criteria result in a limited number of RCTs that fulfill the standards in this study area; hence, these medium-quality studies were chosen. During the data entry phase, the content of all papers will be meticulously reviewed and evaluated to enhance the quality of the meta-analysis.

During the data extraction process, two additional articles were excluded due to insufficient or ambiguous data. The excessive number of sub-variables in both the health dimension and the mindfulness-related psychological component dimension rendered it imprudent to aggregate the effect sizes for meta-analysis. Furthermore, the quantity of certain outcome studies was insufficient for inclusion in the meta-analysis. Following the discussion between the two authors, the health dimension and a part of mindfulness-related psychological component data were removed, resulting in a focus on the three dimensions of mindfulness level, sports performance, and psychological flexibility for discussion (Table 1). Leaving a final set of 18 articles for inclusion in the meta-analysis. These 18 articles reported a total of 34 effect sizes, with eight articles (14 effect sizes, total sample size: 378) investigating the MAIC approach and 10 articles (20 effect sizes, total sample size: 544) examining the MAC approach.

**Table 1:** Quality evaluation.

Rater	Total number of documents	Low quality	Medium quality	High quality	Kappa	*p*
Du	24	3	10	11	0.719	<0.001
Jiang		3	6	15		

**Figure 1:**
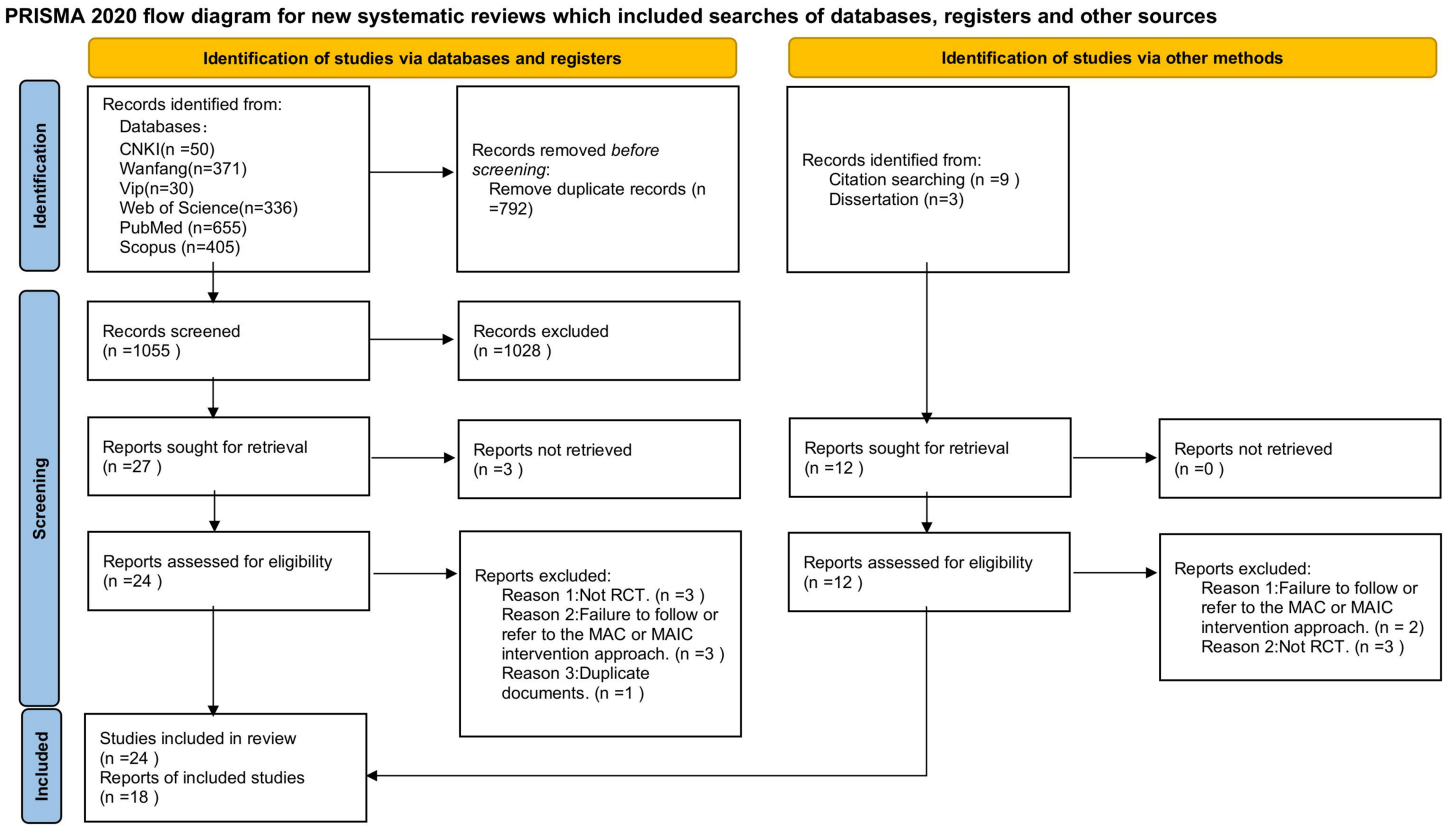
PRISMA 2020 flow diagram.

The study selection process is summarized in a PRISMA (Preferred Reporting Items for Systematic Reviews and Meta-Analyses) flow diagram (Figure 1), which provides a visual representation of the number of studies identified, screened, and included at each stage of the process.

A total of 34 effect sizes were recorded, with eight articles (14 effect sizes, total sample size: 378) in the MAIC category and 10 articles (20 effect sizes, total sample size: 544) in the MAC category. The final set of data provides a solid foundation for the subsequent data analysis and synthesis of the effects of mindfulness interventions on athletes’ outcomes.

### 3.2 Characteristics of included studies

The 18 included studies were published between 2003 and 2023 and were conducted in various countries, including Australia, China, Iran, Sweden, USA. The sample sizes ranged from 18 to 160, with a total of 922 participants across all studies.

The studies investigated the effects of mindfulness training on athletes from a variety of sports, such as badminton, shooting, running, basketball, football, golf, volleyball et al., and included participants of both genders and various skill levels (e.g., novice, intermediate, elite).

The mindfulness interventions employed in the studies were either the MAC approach (*k* = 10) or the MAIC approach (*k* = 8), with intervention durations ranging 30 min, 7 weeks and 8 weeks. The control groups in these studies received either no intervention, waiting list control, or an alternative intervention. Outcome measures included mindfulness, sports performance, health (physical and mental), and mindfulness-related psychological components, assessed using various self-report questionnaires and performance tests (Table 2).

**Table 2:** Input data.

Document coding	Intervention content	Dimensions	Tool	Approach	Duration	Sample	Gender	Sports	Exercise level	Area
(Bu et al., 2020)-1	Mindfulness	M	FFMQ	MAIC	7 weeks	49	M = 29 W = 20	Badminton	High	China
(Bu et al., 2020)-2	Psychological flexibility	P	AAQ-2							
(Liu and Zhang, 2022)-1	Mindfulness	M	FFMQ	MAIC	7 weeks	64	M = 32 W = 32	Mix	Middle	China
(Gang, 2023)-1	Mindfulness	M	FFMQ	MAIC	7 weeks	24	M	Shooting	Middle	China
(Gang, 2023)-2	Psychological flexibility	P	AAQ-2							
(Song and Zhang, 2020)-1	Mindfulness	M	FFMQ	MAIC	7 weeks	74	Mix	Mix	Middle	China
(Song and Zhang, 2020)-2	Sports performance	S	Performance							
(Wang et al., 2021)-1	Mindfulness	M	FFMQ	MAIC	8 weeks	60	W	Long distance running	Low	China
(Zhang et al., 2016)-1	Mindfulness	M	FFMQ	MAIC	8 weeks	43	Mix	Darts	Low	China
(Zhang et al., 2016)-2	Psychological flexibility	P	AAQ-2							
(Zhang et al., 2016)-3	Sports performance	S	Performance							
(Yan et al., 2018)-1	Sports performance	S	Performance	MAIC	7 weeks	40	M	Basketball	Middle	China
(Shen, 2022)-1	Psychological flexibility	P	AAQ-2	MAIC	8 weeks	24	M	Volleyball	High	China
(Shen, 2022)-2	Mindfulness	M	FFMQ							
(Liu et al., 2021)-1	Mindfulness	M	FFMQ	MAC	30 min	57	M = 39 W = 18	Mix	Middle	China
(Naderi et al., 2020)-1	Trait mindfulness	M	CHIME	MAC	7 weeks	160	M	Football	High	Iran
(Naderi et al., 2020)-2	State mindfulness	M	TMS							
(Ajilchi et al., 2022)-1	Mindfulness	M	MIS	MAC	7 weeks	42	W	Unknown	High	Iran
(Dehghani et al., 2018)-1	Sports performance	S	Performance	MAC	8 weeks	31	W	Basketball	Middle	Iran
(Dehghani et al., 2018)-2	Psychological flexibility	P	AAQ							
(Gross et al., 2018)-1	Psychological flexibility	P	AAQ-2	MAC	7 weeks	18	W	Basketball	Middle	USA
(Gross et al., 2018)-2	Mindfulness	M	MAAS							
(Gross et al., 2018)-3	Sports performance	S	Performance							
(Josefsson et al., 2019)-1	Mindfulness	M	AMQ	MAC	7 weeks	69	M = 36 W = 33	Mix	High	Sweden
(Josefsson et al., 2019)-2	Sports performance	S	Performance							
(Macdougall et al., 2019)-1	Mindfulness	M	CAMS-R	MAC	8 weeks	18	M = 5 W = 13	Mix	High	Australia
(Macdougall et al., 2019)-2	Psychological flexibility	P	AAQ-2							
(Macdougall et al., 2019)-3	Overall Performance	S	Performance							
(Mohebi et al., 2022)-1	Mindfulness	M	MIS	MAC	7 weeks	40	W	Mix	High	Iran
(Zadeh et al., 2019)-1	Mindfulness	M	MMPQ	MAC	7 weeks	44	M	Football	Middle	Iran
(Zadeh et al., 2019)-2-1	Performance-Team	S	Performance							
(Zadeh et al., 2019)-2-2	Performance-Individual	S	Performance							
(Perry et al., 2017)-1	Mindfulness	M	FFMQ	MAC	30 min	65	M = 33 W = 32	Golf	Low	USA
(Perry et al., 2017)-2	Sports performance	S	Performance							

M, mindfulness; P, psychological-flexibility; S, sports-performance; AAQ, Acceptance and Action Questionnaire; AMQ, Athlete Mindfulness Questionnaire; CAMS-R, Cognitive and Affective Mindfulness Scale-Revised; CHIME, Comprehensive Inventory of Mindfulness Experiences; FFMQ, Five Facets Mindfulness Questionnaire; MAAS, Mindful Attention Awareness Scale; MIS, Minfulness Inventory for Sport; MMPQ, Mindful Movement Performance Questionnaire.

### 3.3 Assessment of heterogeneity

**Figure 2:**
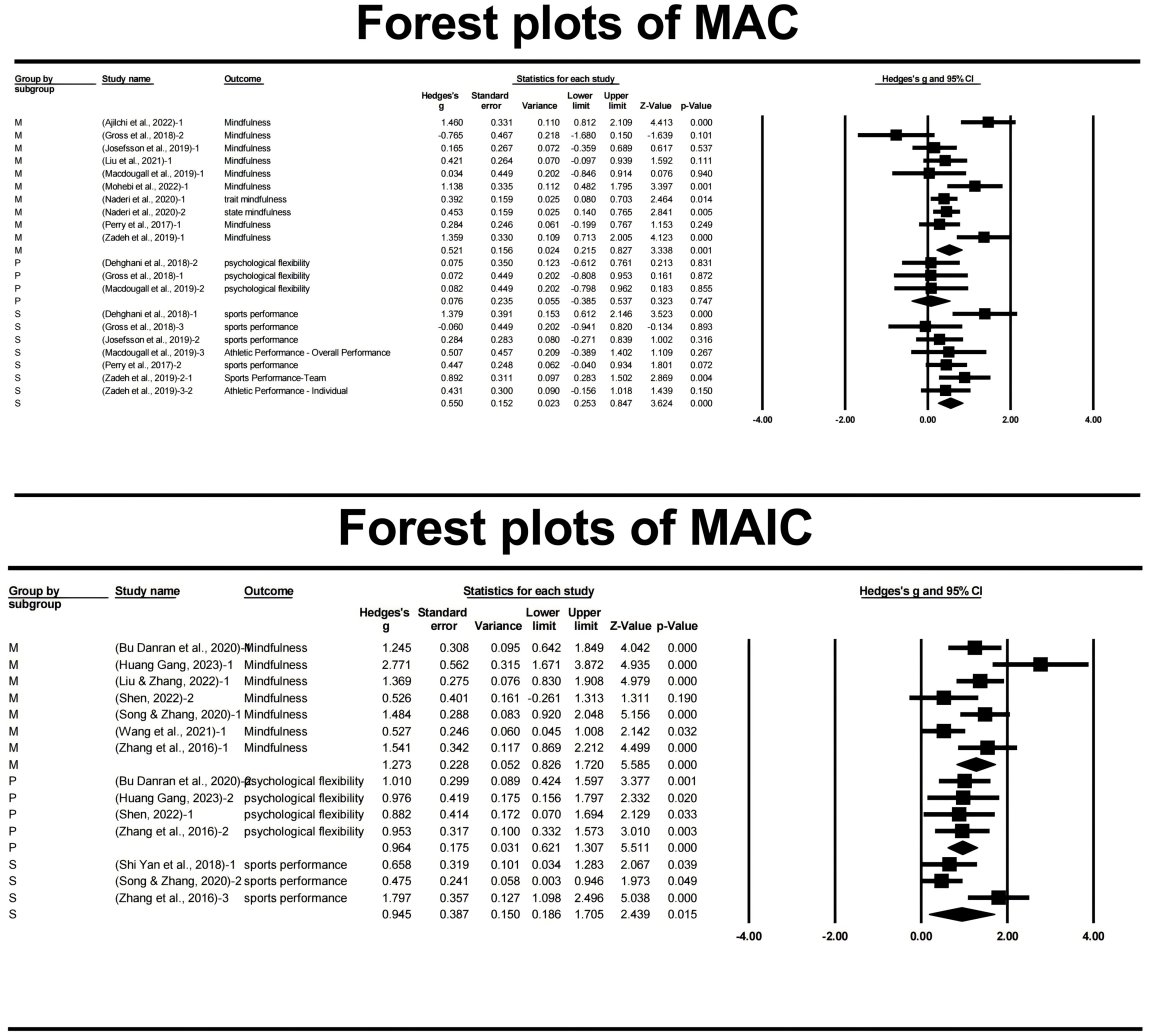
Forest plot.

Heterogeneity tests were conducted separately for the MAC and MAIC studies to assess the variability in effect sizes across the included studies (Figure 2). The *Q* statistic, which evaluates the null hypothesis that all studies share a common effect size, was performed for each subgroup. The results of the *Q* statistic were statistically significant for both MAC (*Q* = 42.208, *df* = 19, *p* = 0.002) and MAIC (*Q* = 33.611, *df* = 13, *p* = 0.001) studies, indicating the presence of heterogeneity in the effect sizes within each subgroup.

To quantify the extent of heterogeneity, the *I^2^* statistic was calculated. The *I^2^* statistic represents the percentage of total variation in effect sizes that can be attributed to true heterogeneity rather than chance. For MAC studies, the *I^2^* value was 54.982%, suggesting moderate heterogeneity. In contrast, the *I^2^* value for MAIC studies was 61.323%, indicating moderate to high heterogeneity.

To investigate the key factors affecting its heterogeneity, a subgroup analysis was conducted to examine the heterogeneity across three dimensions. Mindfulness: MAC (*Q* = 30.238, *df* = 9, *p* < 0.001, *I^2^* = 70.236), MAIC (*Q* = 20.492, *df* = 6, *p* = 0.002, *I^2^* = 70.720); sports performance: MAC (*Q* = 8.758, *df* = 6, *p* = 0.118, *I^2^* = 31.491), MAIC (*Q* = 9.819, *df* = 2, *p* = 0.007, *I^2^ =* 79.631); psychological flexibility: MAC (*Q* < 0.001, *df* = 2, *p* = 1.00, *I^2^ =* 0), MAIC (*Q* = 0.065, *df* = 3, *p* = 0.996, *I^2^ =* 0). When combining effect sizes, both MAC and MAIC have moderate heterogeneity. Following subgroup analysis, the heterogeneity of mindfulness and exercise performance remains substantial, necessitating further investigation into its sources. However, the results concerning psychological flexibility exhibit homogeneity, indicating that psychological flexibility is the most significant contributor to heterogeneity when aggregating effect sizes (Table 3). The restricted quantity of available data precluded a robust explanation in the further analysis of the sources of variability between mindfulness and sports performance.

**Table 3:** Investigation of heterogeneity.

	Group	*Q*	*df*	*p*	*I^2^*
MAC	Mindfulness	30.238	9	<0.001	70.236
	Sport	8.758	6	0.188	31.491
	Psychological	<0.001	2	<1	<0.001
	Total	42.208	19	0.002	54.985
MAIC	Mindfulness	20.492	6	0.002	70.720
	Sport	9.819	2	0.007	79.631
	Psychological	0.065	3	0.996	<0.001
	Total	33.611	13	0.001	61.323
Total		103.961	33	<0.001	68.257

Given the presence of heterogeneity within and across subgroups, the use of a random-effects model for the meta-analysis is justified. The random-effects model accounts for both within-study and between-study variability, providing a more conservative estimate of the overall effect size and allowing for generalizability of the findings to a broader population of studies.

### 3.4 Sensitivity analysis

To assess the robustness of the meta-analysis results and investigate the influence of individual studies on the overall effect sizes, sensitivity analyses were performed using the one-study-removed method. This approach involves iteratively removing one study at a time from the analysis and recalculating the overall effect size and associated statistics.

**Figure 3:**
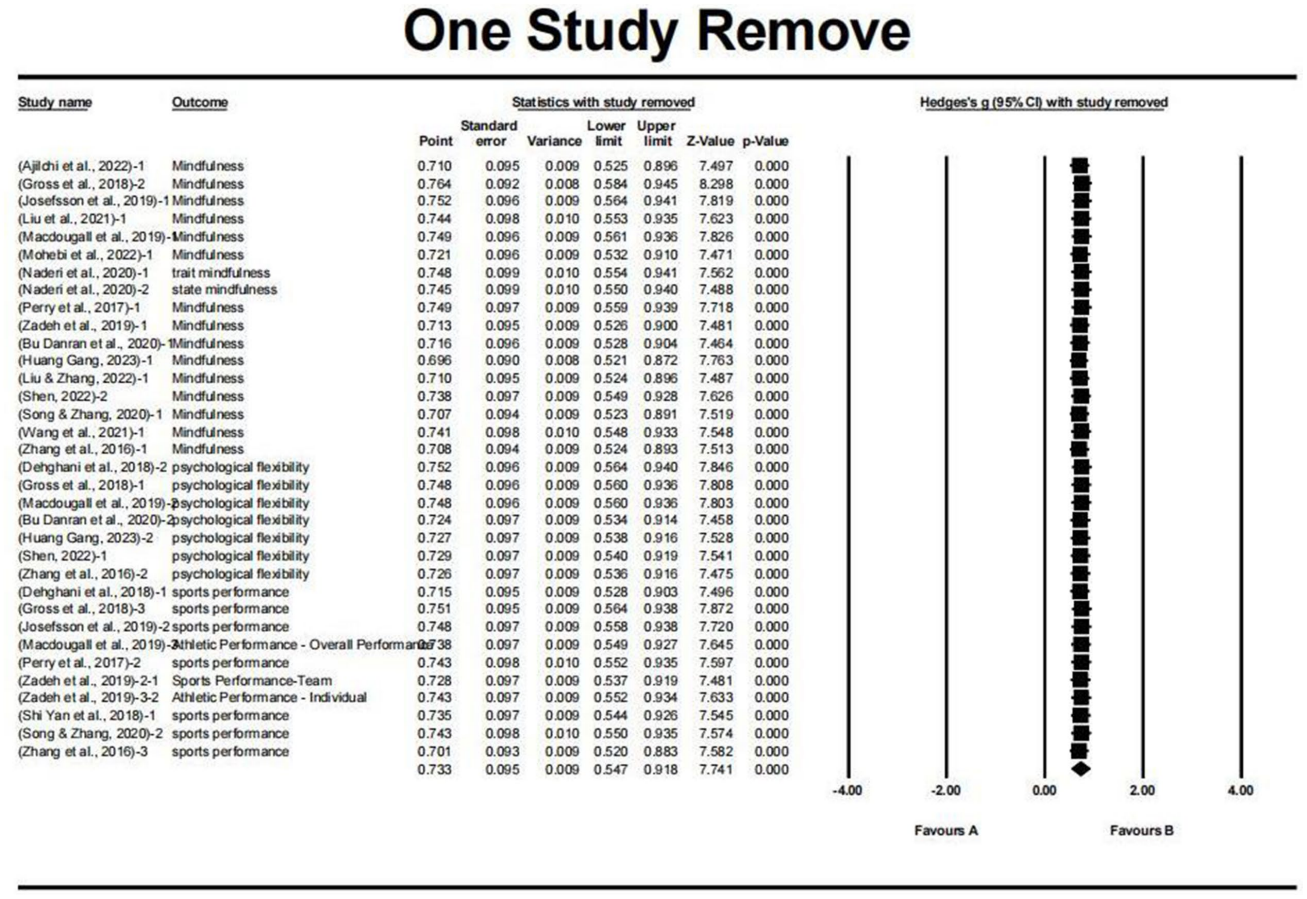
One study remove.

The sensitivity analyses were conducted for the MAC and MAIC. The results of the sensitivity analyses are presented in Figure 3, which display the overall effect sizes and confidence intervals obtained after removing each study in turn.

For both MAC and MAIC, the sensitivity analyses demonstrated that the overall effect sizes remained relatively stable and statistically significant, with no substantial changes observed when individual studies were sequentially removed. This finding suggests that the meta-analysis results are robust and not unduly influenced by any single study (Figure 3).

### 3.5 Publication bias

Publication bias, which refers to the selective publication of studies with significant or positive results, can lead to an overestimation of the true effect size in meta-analyses. To assess publication bias, funnel plots and Egger’s linear regression test were employed.

**Figure 4:**
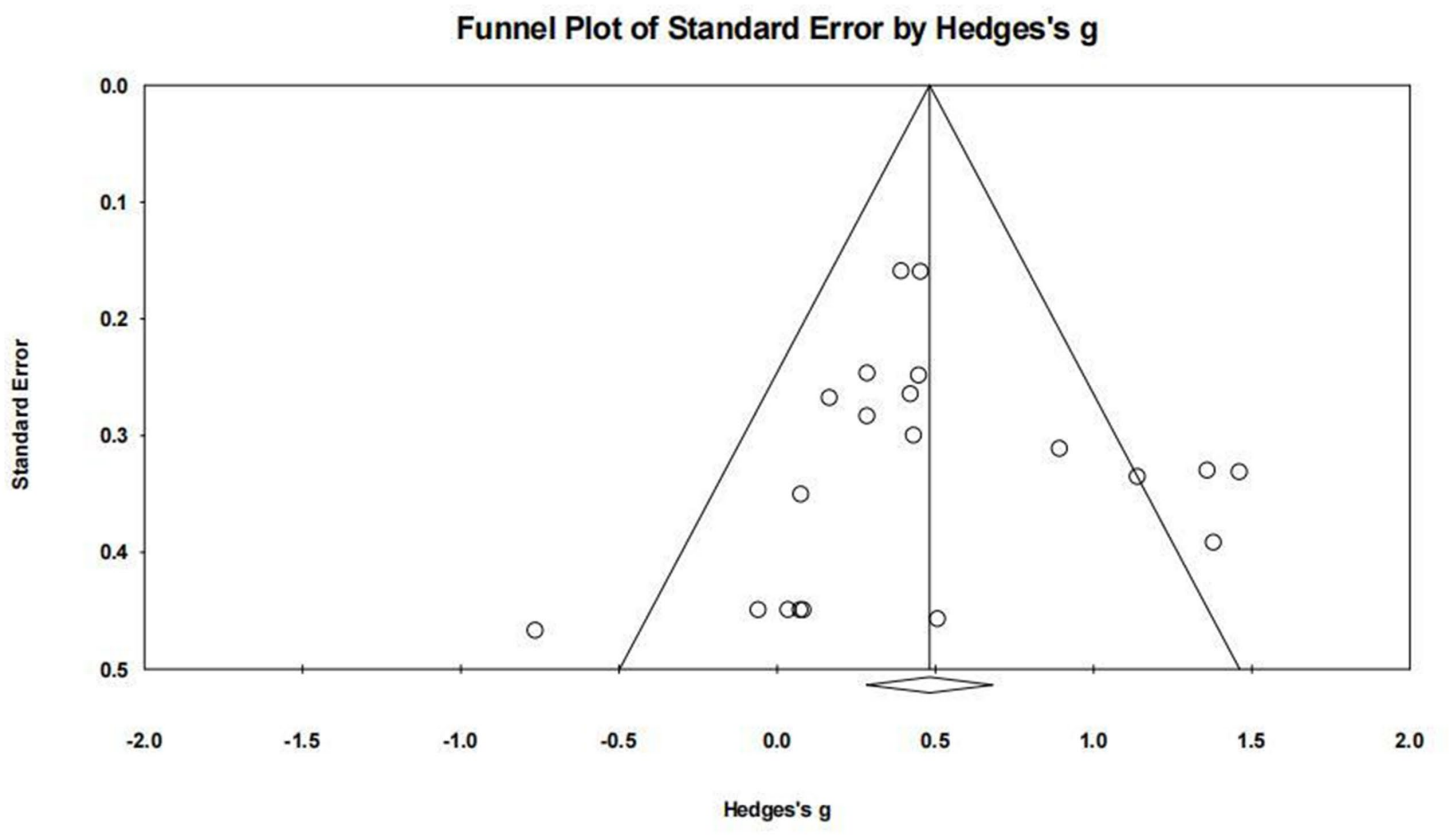
Funnel plot of MAC.

**Figure 5:**
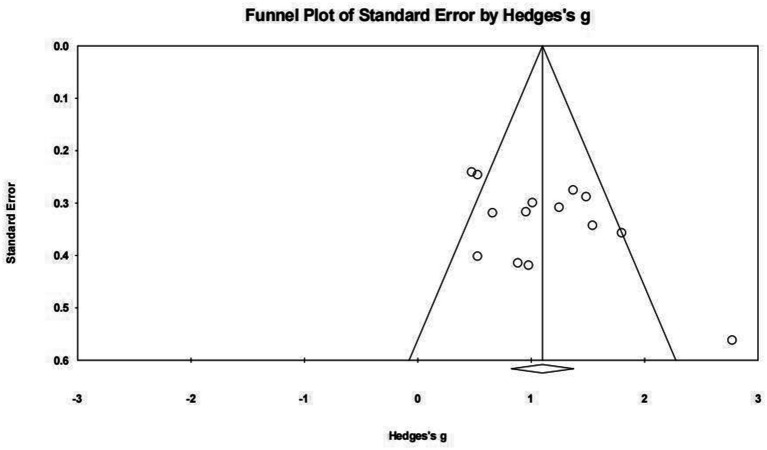
Funnel plot of MAIC.

Funnel plots for the MAC and MAIC subgroups were constructed, displaying the effect size against a measure of study precision. Both MAC and MAIC funnel plot showed a symmetrical distribution, suggesting the absence of substantial publication bias (Figures 4, 5).

Egger’s test quantified the symmetry observed in the funnel plots. For the MAC and MAIC, the test yielded a non-significant result MAC: (intercept = −0.043, 95% CI [−2.188, 2.101], *p* = 0.967 > 0.05); MAIC: (intercept = 3.905, 95% CI [−0.183, 7.992], *p* = 0.059 > 0.05) confirming the absence of significant publication bias (Table 4).

**Table 4:** Egger’s linear regression.

Egger’s regression intercept		
	MAC	MAIC
Intercept	−0.04341	3.90454
Standard error	1.02060	1.87591
95% lower limit (2-tailed)	−2.18762	−0.18272
95% upper limit (2-tailed)	2.10079	7.99180
*t*-value	0.04254	2.08141
*df*	18	12
*p*-value (2-tailed)	0.96654	0.05947

### 3.6 Meta-analysis results

#### 3.6.1 MAC studies

A total of 20 effect sizes from 10 MAC studies were meta-analyzed, yielding a statistically significant overall effect size of (*g* = 0.482, 95% CI [0.282, 0.681], *Z* = 4.736, *p* < 0.001). This result indicates that MAC interventions had a moderate positive effect on the outcomes of interest, with the 95% confidence interval not including zero, further supporting the significance of the finding.

#### 3.6.2 MAIC studies

The meta-analysis of 14 effect sizes from 8 MAIC studies also revealed a statistically significant overall effect size of (*g* = 1.099, 95% CI [0.824, 1.374], *Z* = 7.836, *p* < 0.001). This finding suggests that MAIC interventions had a large positive effect on the outcomes of interest, with the 95% confidence interval not including zero, confirming the significance of the result.

### 3.7 Subgroup analyses

Subgroup analyses were conducted to examine the effectiveness of mindfulness interventions across different outcome domains: mindfulness, sports performance and psychological flexibility.

#### 3.7.1 Mindfulness

Both MAC (*g* = 0.521, 95% CI [0.215, 0.827], *Z* = 3.338, *p* = 0.001) and MAIC (*g* = 1.273, 95% CI [0.826, 1.720], *Z* = 5.585, *p* < 0.001) interventions demonstrated significant positive effects on mindfulness outcomes. However, the effect size for MAIC studies was considerably larger than that for MAC studies, suggesting that MAIC may be more effective in enhancing mindfulness in Chinese athletes (Table 5).

**Table 5:** Data summary.

Group	Subgroup	Number of studies (*k*)	Hedges’ *g*	95%CI	*Z*	*p*
MAC	Mindfulness	10	0.521	0.215–0.827	3.338	0.001
	Sport	7	0.550	0.253–0.847	3.624	<0.001
	Psychological	3	0.076	−0.385 to 0.537	0.323	0.747
	Total	20	0.482	0.282–0.681	4.736	<0.001
MAIC	Mindfulness	7	1.273	0.826–1.720	5.585	<0.001
	Sport	3	0.945	0.186–1.705	2.439	0.015
	Psychological	6	0.964	0.621–1.307	5.511	<0.001
	Total	14	1.099	0.824–1.374	7.836	<0.001
Total		34	0.733	0.547–0.918	7.741	<0.001

#### 3.7.2 Sports performance

MAC (*g* = 0.550, 95% CI [0.253, 0.847], *Z* = 3.624, *p* < 0.001) and MAIC (*g* = 0.945, 95% CI [0.186, 1.705], *Z* = 2.439, *p* = 0.015) interventions both showed significant improvements in sports performance, with moderate effect sizes. Although the effect size for MAIC studies was larger, the difference between MAC and MAIC was less pronounced for this outcome domain compared to other domains (Table 5).

#### 3.7.3 Psychological flexibility

There exists a significant disparity between MAC and MAIC concerning psychological flexibility. MAC (*g* = 0.076, 95% CI [−0.385, 0.537], *Z* = 0.323, *p* = 0.747) did not yield compelling evidence for enhancement in psychological flexibility; however, MAIC (*g* = 0.964, 95% CI [0.621, 1.307], *Z* = 5.511, *p* < 0.001) was statistically significant. The heterogeneity analysis indicates that this dimension is the most important source of variation between the two mindfulness approaches (Table 5).

The subgroup analyses revealed consistent positive effects of mindfulness interventions across mindfulness and sports performance domains for both MAC and MAIC studies. However, the effect sizes for MAIC studies were consistently larger than those for MAC studies. These findings suggest that the culturally-adapted MAIC approach may be more effective than the standard MAC approach in enhancing various outcomes among Chinese athletes. However, the two methodologies produced markedly distinct outcomes on psychological flexibility. This is the most significant distinction between the two approaches (Table 5).

### 3.8 Moderator analyses

Moderator analyses were conducted to explore potential factors that may influence the effectiveness of mindfulness interventions, including gender, intervention duration, and skill level.

#### 3.8.1 Gender

The overall effect size for male athletes (*g* = 0.520, 95% CI [0.017, 1.022], *Z* = 2.028, *p* = 0.043 < 0.05) was slightly larger than that for female athletes (*g* = 0.804, 95% CI [0.495, 1.112], *Z* = 5.111, *p* < 0.001), suggesting that mindfulness interventions may be more beneficial for male athletes. However, both male and female athletes demonstrated significant improvements following mindfulness training.

#### 3.8.2 Intervention duration

The effect sizes increased with longer intervention durations, with 30-min interventions showing a small effect size (*g* = 0.382, 95% CI [0.096, 0.668], *Z* = 2.619, *p* = 0.009), 7-week interventions demonstrating a moderate effect size (*g* = 0.773, 95% CI [0.520, 1.025], *Z* = 5.997, *p* < 0.001), and 8-week interventions exhibiting a large effect size (*g* = 0.776, 95% CI [0.419, 1.134], *Z* = 4.261, *p* < 0.001). These findings suggest that longer mindfulness interventions may be more effective in promoting positive outcomes among athletes.

#### 3.8.3 Skill level

The effect sizes decreased with increasing skill levels, with novice athletes showing a large effect size (*g* = 0.879, 95% CI [0.418, 1.340], *Z* = 3.736, *p* < 0.001), intermediate athletes demonstrating a moderate effect size (*g* = 0.761, 95% CI [0.419, 1.102], *Z* = 4.360, *p* < 0.001), and elite athletes exhibiting a moderate effect size (*g* = 0.627, 95% CI [0.390, 0.865], *Z* = 5.183, *p* < 0.001). This finding suggests that mindfulness interventions may be more beneficial for athletes with lower skill levels, possibly due to the greater room for improvement in their psychological and performance outcomes.

## 4 Discussion

The present meta-analysis aimed to validate the efficacy of the Mindfulness-Acceptance-Commitment (MAC) approach and its Chinese counterpart, the Mindfulness-Acceptance-Insight-Commitment (MAIC) approach, in enhancing mindfulness, sports performance and psychological flexibility among athletes. Additionally, the study sought to compare the two approaches and investigate the importance of cultural adaptations in mindfulness interventions. When comparing the overall effect sizes between the two approaches, the MAIC studies demonstrated substantially larger effects than the MAC studies (Table 5). This finding indicates that the culturally-adapted MAIC approach may be more effective than the standard MAC approach for Chinese athletes, highlighting the potential benefits of considering cultural factors when designing and implementing mindfulness interventions.

The subgroup analyses revealed that MAIC interventions yielded larger effect sizes than MAC interventions in all outcome domains. These findings highlight the potential advantages of incorporating cultural considerations and adaptations when designing and implementing mindfulness interventions for specific populations. The larger effect sizes observed for MAIC studies across different outcome domains highlight the potential benefits of considering cultural factors when designing and implementing mindfulness interventions. The cultural adaptation of mindfulness training may help to better address the specific needs and preferences of Chinese athletes, leading to more pronounced improvements in mindfulness, sports performance.

These subgroup analyses provide valuable insights into the differential effectiveness of MAC and MAIC interventions across various domains, underscoring the importance of cultural adaptation in optimizing the benefits of mindfulness training for specific populations. Future research should further explore the mechanisms underlying the superior effects of MAIC and investigate the potential moderators and mediators of the relationship between culturally-adapted mindfulness interventions and athlete outcomes.

Despite the disparities between MAC and MAIC in mindfulness and sport performance, both outcomes were positive. The heterogeneity analysis indicated that psychological flexibility was the most substantial distinction between the two. The disparities in mindfulness and exercise performance may arise from various factors, including distinct intervention populations, assessment instruments, and exercise modalities. Insufficient data and a dearth of pertinent literature render a robust explanation for the partition of subvariables unattainable. I hope that additional pertinent studies will arise in the future to enhance this information.

The MAC program helps athletes find their core values and develop “value-driven behavior” to solve problems. In essence, it is a value that highlights individualism. This behavior does not solve the relationship between self and society. It is also difficult to integrate oneself into the process and environment (Bu and Si, 2014). Under China’s national system that pursues collectivism, the interests of the country and the collective are above all else. The way of self-expression emphasized by MAC is prone to cultural shock when encountering China’s unique national system. The newly incorporated “Insight” component amalgamates Chinese socio-cultural elements, including adversity coping through acceptance, socially oriented ideals, and insights. It adeptly incorporates Eastern values, plays a very good role in neutralizing the relationship between the individual and the collective, it considers the collectivistic nature of Chinese culture and the importance of social harmony (Si and Jiang, 2011).

The disparity in psychological flexibility between MAIC and MAC may be ascribed to the incorporation of the “Insight” component, which is a kind of non-attachment to thinking, looking at problems from a new perspective, and flexibly using different values and worldview to creatively understand things (Si et al., 2014) it can promotes flexibility in perception, which emphasizes the cultivation of dialectical thinking and aligns with the Eastern philosophical underpinnings of mindfulness (Öllinger and Knoblich, 2009; Su et al., 2019; Si et al., 2014), may be particularly beneficial in promoting psychological flexibility among Chinese athletes. This aligns with the growing recognition of the importance of cultural considerations in the development and delivery of psychological interventions (Castro et al., 2010; Sue et al., 2009). However, the preceding article just indicates that the evidence supporting the efficacy of cultural adaptation is encouraging, and our discussion within the domain of sports is groundbreaking, it effectively illustrates the efficacy of cultural adaptation in mindfulness training approaches. By integrating culturally-relevant concepts and practices, MAIC may facilitate a deeper understanding and internalization of mindfulness skills among Chinese athletes, leading to greater improvements in mindfulness levels, sports performance. It not only makes the MAIC approach more suitable for local athletes, but also indirectly strengthens the athletes’ psychology flexibility, which is the result of cultural adaptation.

The moderator analyses provided valuable insights into the factors that may influence the effectiveness of mindfulness interventions for athletes. The finding that male athletes demonstrated slightly larger effect sizes than female athletes suggests potential gender differences in the receptiveness to and benefits derived from mindfulness training. This may be related to variations in coping strategies, emotional regulation, or socialization processes between genders (Kang et al., 2018; Wang et al., 2020). However, it is important to note that both male and female athletes benefited significantly from mindfulness interventions, highlighting the universal applicability of these approaches.

The positive association between intervention duration and effect sizes underscores the importance of providing athletes with sufficient time and practice to develop and internalize mindfulness skills. Longer interventions may allow for a more comprehensive understanding and application of mindfulness techniques, leading to greater improvements in athletic well-being and performance. This finding aligns with previous research suggesting that the benefits of mindfulness training may be cumulative and require consistent practice (Wu et al., 2013). However, there is still controversy about the intervention length. Some research indicates that even 30-min mindfulness training may yield superior outcomes, with variations in intervention durations not being statistically significant (Carmody and Baer, 2009; Fincham et al., 2023; Ribeiro et al., 2018; Strohmaier et al., 2021). Therefore, more evidence is needed regarding the duration of intervention.

The observation that athletes with lower skill levels demonstrated larger effect sizes compared to elite athletes suggests that mindfulness interventions may be particularly beneficial for novice and intermediate athletes. This may be due to the greater potential for improvement in their psychological and performance outcomes, as they may have had less exposure to mental training techniques compared to elite athletes. However, it is important to consider that elite athletes may face unique pressures and demands that could influence their responsiveness to mindfulness interventions (Birrer et al., 2012). Future research should examine the specific needs and challenges of elite athletes in relation to mindfulness training.

The moderator analyses provide practitioners with valuable information to tailor mindfulness interventions based on athlete characteristics and training contexts. For example, coaches and sport psychologists may consider implementing longer mindfulness programs, particularly for male athletes and those with lower skill levels, to optimize the benefits of mindfulness training. The moderator analyses suggest that gender, intervention duration, and skill level may influence the effectiveness of mindfulness interventions for athletes. These findings can inform the design and implementation of future mindfulness programs in sport settings, allowing for a more targeted and evidence-based approach to enhancing athlete mentally and physically.

The current meta-analysis advances the understanding of the effectiveness of mindfulness interventions in sport contexts and highlights the importance of cultural adaptations. The findings support the notion that incorporating culturally-relevant concepts, practices, and values can enhance the acceptability, relevance, and impact of mindfulness interventions for specific populations (Karl et al., 2022). This underscores the need for researchers and practitioners to consider cultural factors when designing and implementing mindfulness programs in diverse sport settings.

The study’s findings have important practical implications for coaches, sport psychologists, and other professionals working with athletes. The results suggest that mindfulness interventions, particularly those that are culturally adapted, can be valuable tools for promoting athlete mentally and physically. Practitioners should consider incorporating mindfulness training into their repertoire of psychological interventions, tailoring the content and delivery to the specific cultural context and needs of their athletes.

## 5 Limitations

Despite the strengths of this meta-analysis, including the comprehensive literature search, rigorous inclusion criteria, and examination of multiple outcome domains, several limitations should be acknowledged.

First, despite the assured quality of the study, the limited number of studies and sample size will significantly impact the robustness of the results in this meta-analysis, particularly in the MAIC subgroup, suggests that the overall effect sizes may be overestimated, it is hoped that more relevant research can be carried out in the future to enrich its content.

Second, despite our efforts to include only high-quality research, several moderate to low quality researches were incorporated, potentially introducing biases that could compromise the integrity of the meta-analysis results. Numerous articles were lost points in PEDro scale for failing to implement triple blinding (e.g., utilizing only single or double blinding), ambiguous randomization procedures, or inadequate concealment during allocation. Future research needs to concentrate on enhancing the quality of RCTs.

Third, the heterogeneity exists. Although the most significant source of heterogeneity was identified by subgroup analysis, the origin of heterogeneity related to mindfulness and sports performance remains insufficiently clarified. This may be ascribed to differing tools, people, and sporting events, among other factors. We hope to investigate this further in the future.

Fourth, the relatively small number of studies included in some subgroup analyses may limit the generalizability of the findings.

Fifth, the moderator analyses were based on between-study comparisons and may be influenced by other study characteristics or confounding factors. Future research should employ within-study designs to directly compare the effectiveness of mindfulness interventions across different subgroups of athletes while controlling for potential confounding variables.

## 6 Conclusion

This meta-analysis provides compelling evidence for the effectiveness of mindfulness interventions, particularly the culturally-adapted Mindfulness-Acceptance-Insight-Commitment (MAIC) approach, in enhancing various outcomes among athletes. The superior effects of MAIC compared to the standard Mindfulness-Acceptance-Commitment (MAC) approach for Chinese athletes highlight the importance of cultural adaptations in mindfulness interventions.

The subgroup and moderator analyses offer valuable insights into the factors influencing the effectiveness of mindfulness interventions, guiding practitioners in tailoring programs based on athlete characteristics and training contexts. The findings have important practical implications, encouraging professionals to incorporate the cultural adaptation into their transnational movement of practices.

Future research should further explore the mechanisms underlying the differential effects of MAC and MAIC, investigate the long-term sustainability of benefits, and examine the effectiveness of culturally-adapted practices.

## Data Availability

The original contributions presented in the study are included in the article/supplementary material, further inquiries can be directed to the corresponding author.
